# Hybrid gastroenterostomy using a lumen-apposing metal stent: a case report focusing on misdeployment and systematic review of the current literature

**DOI:** 10.1186/s13017-022-00409-z

**Published:** 2022-01-22

**Authors:** Carlo Fabbri, Cecilia Binda, Paola Fugazzola, Monica Sbrancia, Matteo Tomasoni, Chiara Coluccio, Carlo Felix Maria Jung, Enrico Prosperi, Vanni Agnoletti, Luca Ansaloni

**Affiliations:** 1 Gastroenterology and Digestive Endoscopy Unit, Forlì-Cesena Hospitals, AUSL Romagna, Forlì, Italy; 2 General, Emergency and Trauma Surgery Department, M. Bufalini Hospital, Cesena, Italy; 3 Department of Medical and Surgical Sciences, University of Bologna, Sant’Orsola-Malpighi Hospital, Bologna, Italy; 4 Anesthesia and Intensive Care Unit, M. Bufalini Hospital, AUSL Romagna, Cesena, Italy

**Keywords:** Lumen-apposing metal stent, EUS-guided gastroenterostomy, Gastric outlet obstruction, Gastroenterostomy, Complications

## Abstract

**Background:**

Gastric outlet obstruction can result from several benign and malignant diseases, in particular gastric, duodenal or pancreatic tumors. Surgical gastroenterostomy and enteral endoscopic stenting have represented effective therapeutic options, although recently endoscopic ultrasound-guided gastroenterostomy using lumen-apposing metal stent (LAMS) is spreading improving the outcome of this condition. However, this procedure, although mini-invasive, is burdened with not negligible complications, including misdeployment.

**Main body:**

We report the case of a 60-year-old male with gastric outlet obstruction who underwent ultrasound-guided gastroenterostomy using LAMS. The procedure was complicated by LAMS misdeployment being managed by laparoscopy-assisted placement of a second LAMS.

We performed a systematic review in order to identify all reported cases of misdeployment in EUS-GE and their management. The literature shows that misdeployment occurs in up to 10% of all EUS-GE procedures with a wide spectrum of possible strategies of treatment.

**Conclusion:**

The here reported hybrid technique may offer an innovative strategy to manage LAMS misdeployment when this occurs. Moreover, a hybrid approach may be valuable to overcome this complication, especially in early phases of training of EUS-guided gastroenterostomy.

**Supplementary Information:**

The online version contains supplementary material available at 10.1186/s13017-022-00409-z.

## Background

Gastric outlet obstruction (GOO) is a potential complication in malignancies of the upper gastrointestinal tract including gastric, duodenal, pancreatic or biliary tumors [1]. Conventionally, surgical gastroenterostomy (SGE) and endoscopic enteral stenting (ES) are common treatment options. However, SGE has higher complication and mortality rates than ES, which on the other hand demonstrates unsatisfactory patency in patients with life expectancy higher than 6 months [2, 3]. In recent years, endoscopic ultrasound-guided gastroenterostomy (EUS-GE) using lumen-apposing metal stent (LAMS) has been introduced in order to overcome these limitations. Nevertheless, technical success is still suboptimal (around 90%) and complication rates are not negligible (9–17%) [4]. This case report shows a hybrid approach for LAMS deployment, in order to reduce LAMS-associated complications. We further lay focus on recent literature on complications in surgical and endoscopic gastrointestinal anastomosis.

## Main text and case presentation

We report the case of a 60-year-old male patient affected by metastatic pancreatic adenocarcinoma who developed symptoms related to GOO (nausea, vomiting). No prior surgical intervention for his oncologic condition or other abdominal problems were performed. During esophagogastroduodenoscopy a duodenal bulb stenosis was diagnosed. Therefore, decision was taken to perform EUS-GE using an electrocautery enhanced (EC)-LAMS 15 × 10 mm (Hot-Axios, Boston Scientific Corp., Marlborough, Massachusetts, USA), which was performed under general anesthesia in the operating theater for logistical reasons.

For stent deployment, endoscopic antegrade freehand technique was used [5]: over a guidewire, a nasocystic tube was passed over the stricture and the jejunum was filled with contrast and methylene blue; under EUS-guidance, the target loop was identified and punctured using a 19 gauge needle, with aspiration of methylene blue confirming correct needle position in the jejunum. While maintaining the target loop in EUS-view, the EC-LAMS was deployed. However, LAMS release was complicated by misdeployment of the first flange which opened in the lesser sac, probably due to lack of penetration by the EC-LAMS cystotome into the jejunum.

**Fig. 1:**
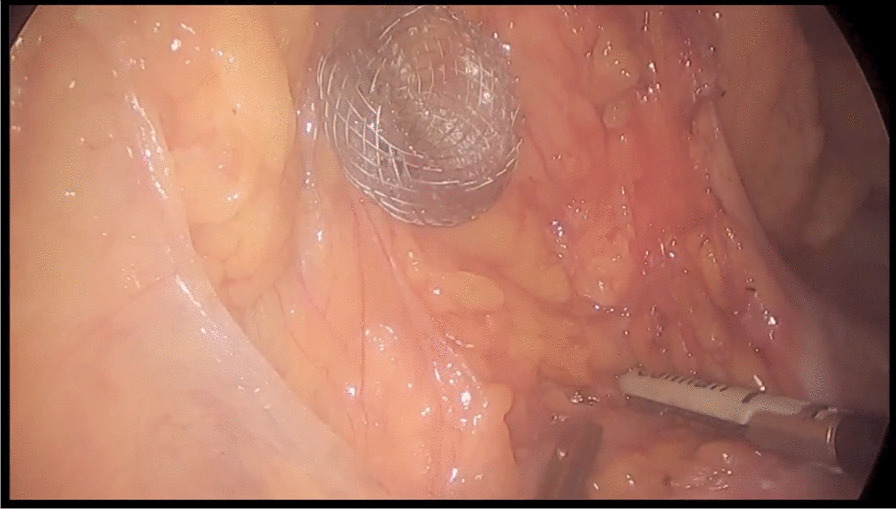
Misdeployment: view of the EC-LAMS opened on the posterior gastric wall

As the endoscopic procedure was performed in an operating room, the chance of immediate exploratory laparoscopy was given. Three laparoscopic trocars were placed (one 10 mm supraumbilical trocar and two 5-mm trocars in the right and left upper quadrants), the gastrocolic ligament was sectioned and the lesser sac explored. The first flange of the stent was found open outside of the posterior gastric wall (Fig. 1), leaning against the transverse mesocolon. Jejunal and transvers colonic perforations were excluded.

**Fig. 2:**
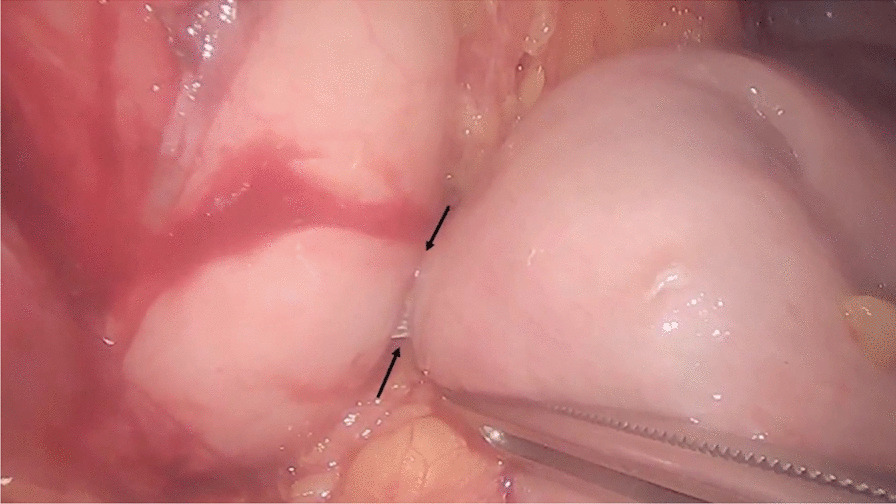
Hybrid technique: laparoscopic view of gastroenteroanastomosis with the EC-LAMS

EC-LAMS was removed endoscopically. Then, laparoscopically, the first jejunal loop after the ligament of Treitz was identified and placed near the stomach. With laparoscopic guidance, we endoscopically released a second EC-LAMS 15 × 10 mm through the previous fistulous gastric tract, performing a laparoscopy-assisted gastroenterostomy (GE). A secure apposition of the LAMS was finally obtained, correct deployment was confirmed both endoscopically and laparoscopically (Fig. 2).

Procedure time for laparoscopy was 95 min and EUS-guided anastomosis via LAMS deployment needed 6 min. No intraprocedural adverse events occurred (Additional file 1: Video S1).

Seventy-two hours after the procedure oral nutrition was initiated and the patient was discharged on postoperative day 7.

## Discussion and conclusions

Establishing gastrointestinal anastomoses is a relatively new endoscopic procedure implemented in 2012 by Binmoeller and Itoi et al. using covered double-anchored metal stents placed via endoscopic ultrasound guidance [6, 7], it rapidly achieved acceptance as a valued alternative for SGE as it was proven to be effective, less invasive and associated with less procedure-related morbidity and mortality. Since 2012, foremost case reports or small case series have been published. Recently, two randomized controlled trials comparing endoscopic vs. surgical GE were published [8, 9] A study by Perez-Miranda et al. study of Perez-Miranda et al. showed that endoscopic GE was associated with fewer postoperative complications and higher technical success than surgical GE (differences non-statistically significant). In a study by Kashab et al. no significant difference was found between endoscopical and surgical GE for adverse events. Technical success was significantly higher in patients treated with surgical GE. Very recently an international multicenter comparison showed that for patients with gastric outlet obstruction EUS-GE and surgical GE have almost identical technical and clinical success; however, reduced time to oral intake, shorter median hospital stay and lower rate of adverse events suggest that the EUS-guided approach might be preferable [10].

Patients with GOO, in which GE becomes necessary, are usually fragile and prone to high morbidity and mortality due to underlying diseases. Therefore, it is necessary to reduce procedure-related mortality as effectively as possible. Use of LAMS for EUS-GE is still considered an off label indication by the American Society of Gastrointestinal Endoscopy [11]. Technical and clinical success rates are reported to be as high as 93 and 90%, respectively [12]. Complications/adverse events associated with LAMS, including misdeployment, are as high as 12% in recent meta-analyses [4].

Various technical endoscopic approaches for EUS-GE exist [5, 13] and so far, it is not clear which one should be favored in order to reduce rate of complications. Chen et al. compared the “direct puncture” with the “balloon assisted” method in a cohort of 77 patients resulting in comparative results concerning complications, technical and clinical success. Only procedure time has been different favoring the direct approach [14]. The “EPASS”-procedure (EUS-guided double-balloon-occluded gastrojejunostomy bypass), which uses a double-balloon-guided occlusion of the jejunal part which will then be connected to the gastric cavity via LAMS, was recently described as one of the safest approaches [15, 16] because of the stable fixation which thereby helps to avoid malpositioning or unsafe LAMS deployment. As for now, it remains in the hands of the endoscopist, which kind of treatment approach to choose, mostly depending on his own experience.

In recent reviews, reported complications associated with EUS-GE are ranging around 12%, including postinterventional pain, bleeding, stent obstruction, stent migration, peritonitis and LAMS misdeployment [4, 12, 17, 18]. A recent multicenter study by Ghandour et al. reported a total of 9.85% (46/467) stent misdeployments counting for one of the most important complications of EUS-GE [19].

Different technical problems can occur during LAMS deployment. Both the proximal or distal flange can be misdeployed, resulting in gastric or jejunal perforation. Also non-target organ puncture can be part of the misdeployment, such as transversing the mesocolon or the transverse colon itself. No standard strategies to overcome LAMS misdeployment exist, its management is up to clinical expertise of each endoscopist.

In order to identify common problems with LAMS deployment/misdeployment and associated problem-solving strategies, we conducted a systematic literature research.

A literature search up to September 2021 among common databases, including PUBMED, SCOPUS, World of Science (WoS), was performed using the following research terms: “axios, lumen-apposing metal stent, gastroenteric anastomosis, gastroenterostomy.”

Publications were accepted in any format, language or publication status. All retrospective, prospective and randomized controlled studies, case reports and case series on humans were included, while studies on animal models were excluded. Studies not mentioning complications related to endoscopic ultrasound-guided gastroenteroanastomosis were excluded.

**Fig. 3:**
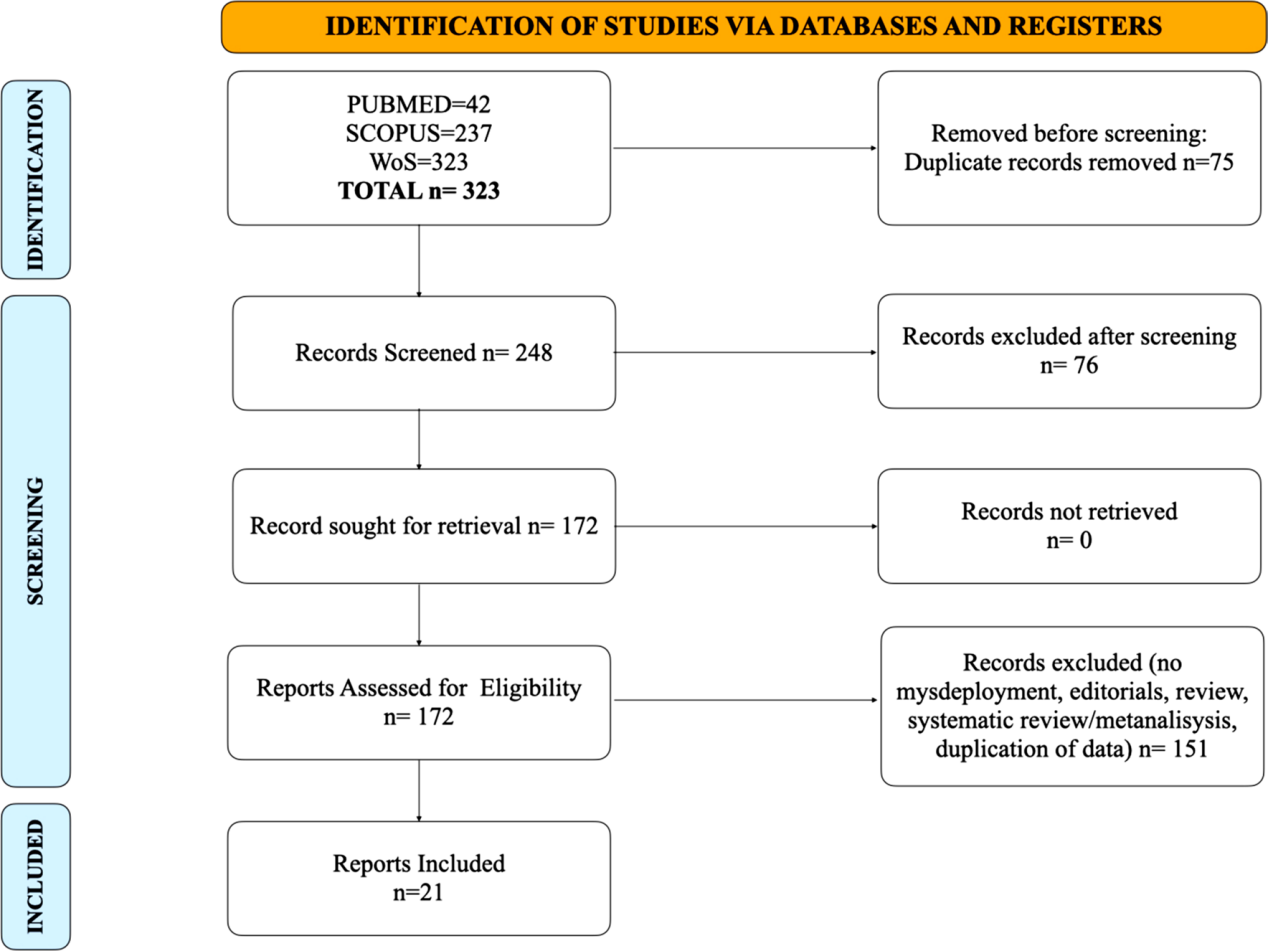
Review flowchart

The initial research identified 323 studies. A total of 75 studies were excluded because of duplicates. Seventy-six were excluded after screening through title and abstract, because not fulfilling the criteria mentioned above. Full text evaluation of 172 studies was fully assessed and included in this systematic review. A total of 151 studies were excluded because of Editorials, review, systematic review with meta-analysis, not reporting misdeployment or duplication of data. Twenty-one studies reported misdeployment (see Fig. 3 and Table 1).

**Table 1:** Publications included in the systematic review

Author	Name of publication	Year of publication	Type of study	EUS-GE technique	Misdeployment	Solution for misdeployment
Bazaga S et al. Endoscopy 2021 [29]	Intraperitoneal endoscopic salvage using an enteral stent for a misdeployed lumen-apposing metal stent during endoscopic ultrasound-guided gastroenterostomy	2021	Case report	Direct = 1	1 distal flange misdeployment into the peritoneal cavity	SEMS stent placement through LAMS
Bejjani M et al. GIE Abstract 2021 [30]	Clinical and Technical Outcomes of patients undergoing EUS-Guided Gastroenterostomy using 20 mm vs 15 mm LAMS	2021	Retrospective	*n* = 267, procedure non-specified	23 (8.6%); 13 in 15 mm LAMS group; 10 in 20 mm LAMS group. No specification concerning flange available	Not specified
Chen Y-I. et al. Surg Endosc 2017 [26]	EUS-guided gastroenterostomy is comparable to enteral stenting with fewer re-interventions in malignant gastric outlet obstruction	2017	Retrospective	Total 30 EPASS = 22; Balloon assisted = 6; Direct = 2	3/30 (10%) misdeployment into the peritoneum	LAMS removal, conservative treatment, one patient requiring surgical therapy for stent removal from the peritoneal cavity
Chen Y.-I. et al., Gastroenterology 2017 [24]	Displaced Endoscopic Ultrasound-Guided Gastroenterostomy Stent Rescued With Natural Orifice Transluminal Endoscopic Surgery	2017	Case report	Direct = 1	1 dislodgement of distal flange into the peritoneum	NOTES exploration of the peritoneal cavity, new LAMS deployment using a gastroscope, through the jejunal puncture Defect
Chen Y.-I. et al., GIE 2018 [14]	EUS-guided gastroenterostomy: a multicenter study comparing the direct and balloon-assisted techniques	2018	Retrospective	Total 77 Direct = 52; Balloon assisted = 22	5 (7%): stent misdeployment into the peritoneum	Immediate Stent replacement (= 4) technique not specified, defect closure (* n* = 1), technique not specified
Colombo M et al., Am J Gastroenterol 2021 [31]	Salvage Procedure for Double Trouble in Lumen-Apposing Metal Stent Misdeployment During Endoscopic Ultrasound-Guided Gastroenterostomy: Ready to Start Again	2021	Case report	Direct = 1	1 dislodgement of distal flange	LAMS removal, gastric perforation closure using an omental fat patch, jejunal leak closure using clips, repeated EUS-GE using direct technique in a distal jejunal loop
Costa Martins et al. VideoGIE 2020 [32]	Lessons learned from a salvage procedure for lumen-apposing metal stent misplacement during EUS-guided gastrojejunal bypass	2020	Case report	EPASS = 1	1, distal flange misdeployment into the peritoneum	NOTES, exploration of the abdominal cavity, Reassembling of the LAMS system; second successful EPASS attempt
Kerdsirichairat et al. Endosc Int Open 2019 [21]	Durability and long-term outcomes of direct EUS-guided gastroenterostomy using lumen-apposing metal stents for gastric outlet obstruction	2019	Retrospective	Direct = 57	2/57 (3.5%); with proximal flange misdeployed in the peritoneum	Immediately retrieved endoscopically and the gastric defects closed with an over-the-scope clip. A new LAMS was then deployed successfully in both cases
Ge PS et al., Surg Endoscopy 2019 [33]	EUS-guided gastroenterostomy versus enteral stent placement for palliation of malignant gastric outlet obstruction	2019	Retrospective analysis of a prospectively collected database	Direct = 22	2/22 (8.3%) misdeployment resulting in perforation, site not specified	LAMS deployment in the same session, and neither case required surgery
Ghandour B., I., EUS-GE Study Group, GIE 2021 [19]	Classification, outcomes and management of misdeployed stents during EUS-guided gastroenterostomy	2021	Retrospective	Total 467; balloon assisted + direct puncture technique used, no information concerning n	46 (9.85%); misdeployment types: I: (distal flange into the peritoneum without enterotomy) = 29 (63.1%); II: (distal flange into the peritoneum despite enterotomy) = 14 (30.4%); III: (distal flange deployed correctly, proximal flange in the peritoneum) = 2 (2.2%); IV: (malpositioning of distal flange in the colon) = 2 (2.2%)	Type I: gastrotomy closure using OTSC/TTSC/Endoscopic suturing/no closure/new LAMS deployment through the same gastrotomy/surgical intervention for peritonitis Type II: new LAMS deployment / Bridging fully covered SEMS through misdeployed LAMS;/NOTES placement of a new LAMS/gastrotomy closure only type III: NOTES retrieval type IV: LAMS removal and fistula closure using TTSC/endoscopic suturing
Gornals J.B. et al. Endoscopy 2021[34]	Helpful technical notes for intraperitoneal natural orifice transluminal endoscopic surgery (NOTES) salvage in a failed EUS-guided gastroenterostomy scenario	2021	Technical paper			(1) if guide wire still in place: new LAMS placement, (2) LAMS in LAMS rescue; (3) NOTES rescue; (4) surgery
Havre RF et al., Scand J Gastroenterol 2021 [35]	EUS-guided gastroenterostomy with a lumen-apposing self-expandable metallic stent relieves gastric outlet obstruction—a Scandinavian case series	2021	Retrospective	Direct = 33	1 distal flange misdeployment into the peritoneum	Gastric fistula closure with clips
Itoi et al. Gut 2016 [16]	Prospective evaluation of endoscopic ultrasonography-guided double-balloon-occluded gastrojejunostomy bypass (EPASS) for malignant gastric outlet obstruction	2016	Prospective	EPASS = 20	2/20 (10%) stent misemployment location unknown	Stent was removed and the patient was treated by conservative therapy
James et al. GIE 2020 [25]	EUS-guided gastroenterol anastomosis as a bridge to definitive treatment in benign gastric outlet obstruction	2020	Retrospective	Total = 22 orojejunal tube-assisted water instillation = 5 (22.7%), balloon-assisted in 8 (36.4%) and fluid instillation with freehand puncture using electrocautery = 9 (40.9%)	1/22 (4.5%) transcolonic misdeployment into the jejunum → no signs of perforation	Patient awaiting surgery
Kouanda et al. Surg Endosc 2021 [36]	Endoscopic ultrasound-guided gastroenterostomy versus open surgical gastrojejunostomy: clinical outcomes and cost effectiveness analysis	2021	Retrospective	Direct = 40	1/40 (2.5%) deployment into the peritoneum,	LAMS removal, defect closure with OTSC, enteral stent placement
Ligresti D et al., Endoscopy 2019 [37]	The lumen-apposing metal stent (LAMS)-in-LAMS technique as an intraprocedural rescue treatment during endoscopic ultrasound-guided gastroenterostomy	2019	Case report	Balloon assisted = 1	1 dislodgement of distal flange into the peritoneum, guide wire still in place in the jejunum	LAMS in LAMS new deployment/bridging
Nguyen NQ et al., Endoscopy 2021 [8, 38]	Endoscopic ultrasound-guided gastroenterostomy using an oroenteric catheter-assisted technique: a retrospective analysis	2021	prospectively collected database Retrospective data analysis	Oroenteric catheter-assisted technique = 42	1 distal flange misdeployment due to failed sheath retraction	Endoscopic suturing of the gastrotomy (Apollo Overstitch)
Perez-Miranda et al. J Clinical Gastroenterol 2017	EUS-guided Gastrojejunostomy Versus Laparoscopic Gastrojejunostomy An international Collaborative Study	2017	Retrospective	Direct = 6, Balloon assisted = 9, Ultraslim endoscope—assisted = 7; Nasobiliary tube assisted = 3	9/25 (36%) localization unknown	Bridging fully covered self-expanding metal stent or a second LAMS* n* = 6, 3 had their LAMS removed and the access site closed with an over-the-scope clip (* n* = 1) or an enteral stent (* n* = 2)
Sondhi AR, Law R, VideoGIE 2020 [39]	Intraperitoneal salvage of an EUS-guided gastroenterostomy using a nested lumen-apposing metal stent	2020	Case report	Direct = 1	1 distal flange misdeployment into the peritoneal cavity	LAMS in LAMS deployment using the same access ecoendoscopically. Finally securing both LAMS with endoscopic sutures
Tyberg et al. Endosc Int Open 2016 [20]	Endoscopic ultrasound-guided gastrojejunostomy with a lumen-apposing metal stent: a multicenter, international experience	2016	Prospective	Total = 26 NOTES = 2, Direct = 3, Balloon assisted = 13, Ultraslim endoscope assisted = 5, Nasobiliary tube assisted = 3	7/26 (26.9%) partial LAMS misdeployment, either proximal or distal flange	Misplacement of the proximal flange beyond the gastric wall: tract bridging with fully covered SEMS in 2 of the 4 patients with distal flange misplacement, tract salvage with NOTES access (1 planned and 1 unplanned) and placement of a bridging LAMS instead of an FCSEMS In the 2 patients with unsalvaged distal flange misplacement, the LAMS was pulled back into the stomach and access site was closed with an over-the-scope clip (* n* = 1) or an enteral SEMS without any attempt at closure (* n* = 1) In 2 additional patients, a bridging FCSEMS was placed despite correct placement of a LAMS because of concerns for delayed migration arising from tenting of the LAMS after deployment
Wannhoff et al. Surg Endosc 2021 [40]	Endoscopic gastrointestinal anastomose with lumen-apposing metal stents: predictors of technical success	2021	Retrospective	Total 35; Direct with cautery = 22, Guidewire assisted* n* = 10 Others = 2	4/35 (11.42%)* n* = 2 dislocation of distal stent flange;* n* = 1 dislocation of proximal stent flange;* n* = 1 unsuccessful puncture of the targeted loop	OTSC closure of gastric wall defect before the second attempt. Jejunal wall defect could not be reached, therefore not occluded

We hereby name the most frequently used strategies according to the initial issue of LAMS misdeployment:

A) LAMS proximal flange misdeployment: The fistulous tract into the jejunum is already established but the proximal flange is misdeployed into the peritoneum and is not anchored in the gastric wall. Through the gastric puncture site, another LAMS or a fully covered metal stent can be placed in order to bridge the already placed LAMS [8, 20]. Alternatively, LAMS can be removed completely, the gastric puncture site closed with an over-the-scope-clip (OTSC, Ovesco, Tübingen, Germany) and a new LAMS placed via a new access [21, 22].
B) LAMS distal flange misdeployment: During puncture of the jejunum, the jejunum dislocates, and the distal flange cannot be opened or is only partially opened into the target site. The distal flange therefore partially remains in the peritoneum creating a free perforation of the gastric wall. Here, either LAMS can be completely removed and a fully covered bridging stent or a second LAMS be inserted [20, 23]. During misdeployment of the distal flange without puncturing the jejunum in two patients reported by Kashab et al., LAMS removal and only conservative treatment were performed. An additional option is to create a NOTES access in which the originally created fistulous tract, created by the LAMS, can be secured endoscopically [20, 24]. In cases where the jejunal wall defect could not be reached by endoscopy, Wannhoff et al. preferred to insert a duodenal fully covered stent to bridge the GOO inducing tumor, whereas the jejunal puncture was not occluded [22]. Interestingly, this did not result in further peritonitis originating from the jejunum.
C) Stent misdeployment perforating other organs such as the mesocolon or the transverse colon is a complication which needs surgical intervention [25].
D) Stent misdeployment into the peritoneal cavity: in rare cases, when LAMS cannot be retrieved endoscopically from the peritoneal cavity, stent removal by abdominal surgery might be necessary [26].

The hybrid technique described in this case report has several significant advantages. Probability of incorrect deployment of the first flange is up to 27% [17]. Therefore, a laparoscopically assisted procedure outperforms the limitation of a 2-dimensional endoscopic exam, in this case the incorrect visualization of the target loop by endoscopic ultrasound and furthermore the lack of correct cystostome penetration of the jejunal wall.

Furthermore, this hybrid approach may have the ability to considerably shorten overall procedure time while securing success of endoscopic LAMS deployment. Mean procedure time for laparoscopic GE varies widely from 75 to 170 min in the literature [27]. In our case, time for laparoscopy was 95 min, which included the exclusion of jejunal and colonic perforations and recovery of the flange of EC-LAMS. However, procedure time for GE by LAMS was only 6 min.

Another advantage of this hybrid approach may be the possibility of performing anastomoses between the posterior gastric wall and the first jejunal loop, therefore maintaining a maximum of intestinal absorption surface and reducing the risk of malabsorption and malnourishment [28].

However, limitations are mainly related to the availability of infrastructure and medical staff. EUS-GE usually is not performed in an operating theater. Moreover, this hybrid approach requires simultaneous involvement of two teams, surgeons and endoscopists, which is uncommon and more costly.

To our knowledge, this clinical case reported is the first to show a combined endoscopic and surgical treatment approach in order to overcome endoscopic restrictions for GE, in particular LAMS misdeployment. In cases where endoscopic orientation is difficult and LAMS deployment therefore is at risk, we propose a combined endoscopic and surgical approach in order to reduce procedure time and provide higher safety standards. Further studies need to confirm this observation.

## Supplementary Information

**Additional file 1. Video S1**: EUS-GE, confirmation of the misdeployment of the EC-LAMS, laparoscopic procedure of the GE using a second EC-LAMS

## Data Availability

All data generated during this study are included in this published article and its supplementary information files. Further minor datasets are available from the corresponding author on reasonable request.

## Abbreviations

ES: Endoscopic enteral stenting
EUS: Endoscopic ultrasound
EUS-GE: Endoscopic ultrasound-guided gastroenterostomy
GE: Gastroenterostomy
SGE: Surgical gastroenterostomy
GOO: Gastric outlet obstruction
LAMS: Lumen-apposing metal stent
